# Monitoring of Dynamic Plantar Foot Temperatures in Diabetes with Personalised 3D-Printed Wearables

**DOI:** 10.3390/s21051717

**Published:** 2021-03-02

**Authors:** Christopher Beach, Glen Cooper, Andrew Weightman, Emma F. Hodson-Tole, Neil D. Reeves, Alexander J. Casson

**Affiliations:** 1 Department of Electrical and Electronic Engineering, The University of Manchester, Manchester M13 9PL, UK; alex.casson@manchester.ac.uk; 2 Department of Mechanical, Aerospace and Civil Engineering, The University of Manchester, Manchester M13 9PL, UK; glen.cooper@manchester.ac.uk (G.C.); andrew.weightman@manchester.ac.uk (A.W.); 3 Research Centre for Musculoskeletal Science & Sports Medicine, Department of Life Sciences, Faculty of Science and Engineering, Manchester Metropolitan University, Manchester M15 6BH, UK; e.tole@mmu.ac.uk (E.F.H.-T.); n.reeves@mmu.ac.uk (N.D.R.)

**Keywords:** 3D printing, diabetes, diabetic foot, diabetic peripheral neuropathy, digital health, personalised medicine, prevention, foot temperature monitoring, telehealth, wearables

## Abstract

Diabetic foot ulcers (DFUs) are a life-changing complication of diabetes that can lead to amputation. There is increasing evidence that long-term management with wearables can reduce incidence and recurrence of this condition. Temperature asymmetry measurements can alert to DFU development, but measurements of dynamic information, such as rate of temperature change, are under investigated. We present a new wearable device for temperature monitoring at the foot that is personalised to account for anatomical variations at the foot. We validate this device on 13 participants with diabetes (no neuropathy) (group name D) and 12 control participants (group name C), during sitting and standing. We extract dynamic temperature parameters from four sites on each foot to compare the rate of temperature change. During sitting the time constant of temperature rise after shoe donning was significantly (*p* < 0.05) faster at the hallux (*p* = 0.032, 370.4 s (C), 279.1 s (D)) and 5th metatarsal head (*p* = 0.011, 481.9 s (C), 356.6 s (D)) in participants with diabetes compared to controls. No significant differences at the other sites or during standing were identified. These results suggest that temperature rise time is faster at parts of the foot in those who have developed diabetes. Elevated temperatures are known to be a risk factor of DFUs and measurement of time constants may provide information on their development. This work suggests that temperature rise time measured at the plantar surface may be an indicative biomarker for differences in soft tissue biomechanics and vascularisation during diabetes onset and progression.

## 1. Introduction

Diabetic foot ulcers (DFUs) are a life-changing complication of diabetes, affecting an estimated 25% of those with diabetes [1]. The condition is complex, costly to treat, and has a large impact on quality of life. In England, the cost of treatment is greater than the combined cost of breast, prostate and lung cancers and the incidence is increasing globally [2]. Worldwide it is estimated that a limb is lost every 30 s as a result of diabetes [3]. Improved foot care and prevention techniques, such as reducing the use of the foot if the patient is at-risk [4], could reduce foot ulceration. For example, Bus et al. [5] estimate that 75% of ulcers are preventable with suitable care and preventative monitoring. Wearable devices are becoming increasingly common for use in preventative medicine in areas such as cardiovascular health [6], motivating the creation of wearables for monitoring risk factors in DFU. In [7,8,9], the authors demonstrate that wearable and other smart devices could reduce the incidence of DFUs by providing continuous long-term monitoring and feedback, which may allow better long-term management of DFUs. Wearable devices can reduce the demand on clinics, the need to travel and the time lag between check-ups [8].

Risk factors to detect DFU formation include peripheral neuropathy (a loss of sensation at the foot), foot deformities, and increased plantar pressures [10,11]. The monitoring of plantar pressures, normal to the foot surface, is commonly used to generate in-clinic evidence of ulcer formation [12,13,14,15], and plantar pressures can be monitored in wearable devices including the SurroSense Rx [16]. For out-of-the-clinic use, Abbott et al. [17] estimate that real-time pressure offloading alerts from a plantar pressure smart insole reduced ulcer recurrence up to 86%. Recent review publications covering the common wearable systems for foot pressure sensing and their performance are given in [18,19] respectively. However, there is much debate on the utility of plantar pressure as a risk factor for DFU [19]. Many works argue that monitoring shear pressure may provide more information on ulcer formation [20,21,22]. Unfortunately, while new sensors are appearing to enable this [23], shear pressure is costly and difficult to measure [24].

To overcome the above limitations, increasingly common in clinical practice is measuring temperature asymmetry between the same site on opposite feet, with a common alert threshold of 2.2 °C [25,26] used to indicate potential DFU risk. For example, the point-of-care Podimetrics SmartMat can alert to the potential onset of ulceration if the 2.2 °C threshold between identical sites on opposite feet is exceeded [27]. The pressing need for integrating this similar functionality into wearable devices has been highlighted by Golledge et al. [9], who stated the need for more user-friendly sensors to automate home foot temperature monitoring. This need can be achieved by implementing temperature sensing into insole or shoe-based wearable devices; which can provide long-term and continuous measurements of foot temperature. These devices will allow the temperature dynamics of the foot, for example how quickly it heats up when shoes are put on, to be studied for the first time.

To date, only a very limited number of works have investigated temperature sensing wearables for DFU. The Siren Socks [28] are commercially available for wearable foot temperature sensing. However, the monitoring of temperature in these commercial devices has focused on singular, discrete measurements rather than investigating the more complex dynamics of how temperature changes over time [29]. Recently, Niemann et al. [30] investigated plantar temperatures at the foot in participants with diabetes (and neuropathy). They investigated temperature at eight fixed sites on the foot (the hallux, the five metatarsal heads, the lateral and the calcaneus), during repeated standing periods. Niemann et al. identified that plantar temperatures followed a downward trend during standing, and did not find significant differences between those with diabetes against controls. Niemann et al. did not extract any metrics on the temperature dynamics during sitting or standing, and this time-dependent information, which has been under-investigated, may provide more information to detect DFU development sooner. More recently still, Leister et al. [31] investigated temperature dynamics at the foot at a single fixed site during resting, walking and cooling down periods in participants with type 2 diabetes with and without transtibial (below knee) amputation. Leister et al. identified that those without amputation displayed an increased rate of temperature change during walking. Further investigation is needed to build on this work to identify if these differences in the rate of temperature change exist between controls (without diabetes), and those with diabetes prior to any amputation or developing any of the risk factors associated with DFUs, such as peripheral neuropathy.

Here, in this paper, we present a flexible 3D-printed wearable device to monitor continuous foot temperatures and allow personalisation of the sensor placement to account for differences in anatomy. As the main contribution of this paper, we also present a feasibility study using our custom novel device to investigate the temperature dynamics at the plantar foot during sitting and standing, with controls and participants with diabetes, for the first time. These continuous measurements on rates of temperature change may provide more information and thus better prevention of diabetic foot conditions. While previous work by Reddy et al. [32] has investigated temperature rise at the foot during walking in healthy participants, this paper is the first, to the best of the authors’ knowledge, to investigate the temperature rise time at the plantar foot in participants with diabetes compared to controls. This work also considers the personalisation of the device to account for variations in anatomy for the first time. By personalising the sensor positioning for each participant, sensors can be aligned with the anatomy of each participant’s foot, enabling results to be normalised as each sensor is located on identical anatomical locations between participants. This personalisation may improve performance compared to previous devices, which only account for variations in foot size rather than the differences in the internal anatomy within the same foot size. In addition, this personalisation can allow sensing to focus on the most high-risk areas which vary depending on the diabetic foot condition. For example, the high-risk sites for DFU include the hallux and metatarsal heads [21], however, for those with Charcot foot, the midfoot is a more common location for ulceration [33], so it may be desirable to focus sensing around this area. There is considerable interest in personalised and precision medicine in diabetes, where it is seen as a key enabler to improved and more cost-effective treatments [34,35]. 3D-printing is a key tool to enable this, due to its ability to rapidly produce customised devices, and we take advantage of this in our new device. This work presents the creation of a new personalised device to monitor the temperature at the plantar foot and aims to identify if the temperature dynamics at the foot are different in participants with diabetes compared to controls.

## 2. Materials and Methods

### 2.1. Study Device

**Figure 1:**
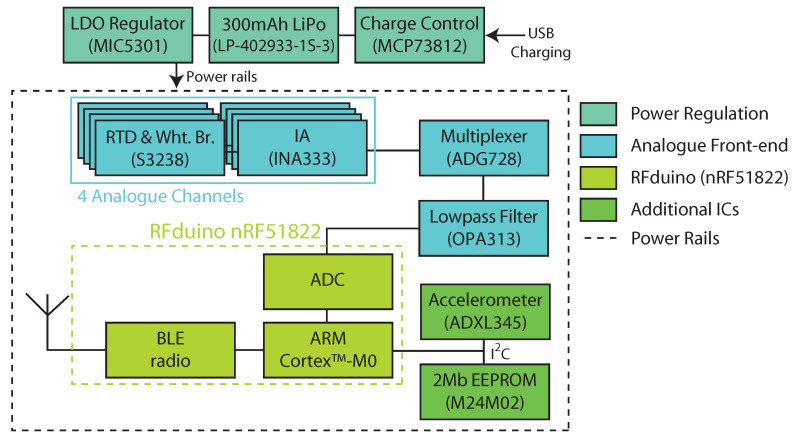
Block diagram of the main electronic components in the smart insole system developed in this work. Lines denote signal flow between components of the schematic. Abbreviations: Low Dropout (LDO), Resistance Temperature Detector (RTD), Wheatstone Bridge (Wht. Br.), Instrumentation Amplifier (IA), Analogue-to-Digital Converter (ADC), Bluetooth Low Energy (BLE), Electrically Erasable Programmable Read-Only Memory (EEPROM).

We fabricated custom circuitry and flexible insoles for use in this study. The circuitry for the wearable device is summarised in Figure 1. This compromises of a four-channel analogue front-end centred around a Wheatstone bridge to interface with flexible platinum Resistance Temperature Detector (RTD) sensors (S3238, Minco Products, Inc., Minneapolis, MN, USA), an instrumentation amplifier (TI INA33), multiplexer (Analog Devices ADG728) and a Sallen–Key low-pass filter (TI OPA313) with a cut-off frequency of 10 Hz. This circuitry interfaces with the 10-bit Analogue-to-Digital Converter (ADC) of an RFduino (based around a Nordic Semiconductor nRF51822). The nRF51822 features an Arm™ Cortex-M0 and Bluetooth Low Energy (BLE) radio, which we make use of here to transmit data from the device to an accompanying smartphone app. Our device also features an accelerometer for activity detection and an 2 Mb EEPROM for long-term data storage, but we do not make use of either of these features in this work. The device is powered from a 300 mAh lithium polymer (LiPo) cell, a 3 V LDO regulator (MIC5301), and a charge controller (MCP73812). The RTD sensors we make use of are developed for industrial applications, such as measuring the temperature of motor windings, a more mechanically challenging environment than the environment the sensors will be exhibited to in this study.

**Figure 2:**
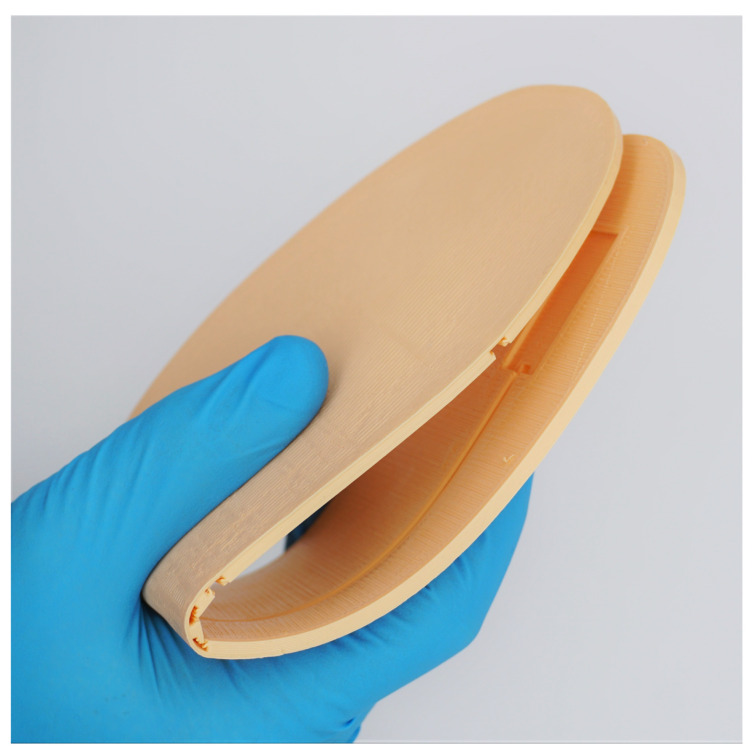
Demonstration of the flexibility of the 3D-printed insoles used in this study.

Custom 3D-printed flexible insoles were created for this study, with a pair provided to each participant. The insoles were printed from flexible thermoplastic polyurethane (TPU) material with shore hardness of 85A (NinjaFlex, Fenner Drives, Inc., Manheim, PA, USA) and containing cut-outs (sized at 12.7 × 31.8 × 1.3 mm) for mounting the RTD temperature sensors. The locations of the sensor mountings were personalised for each participant’s foot, matching sensor positions to the most common locations for ulceration at the foot (the hallux, first and fifth metatarsal heads and calcaneus) [10]. The process for identifying these locations is detailed later in Section 2.3. An example of the flexibility of these insoles is demonstrated in Figure 2.

The insoles were passivated by coating in a layer of 0.3 mm, 30° shore silicone (Translucent Silicone, Silex Silicones Ltd., Hampshire, UK) laser cut to the size of the insole and held in place with double-sided tape. Both the RTD sensors and the 3D-printed insole are flexible, to improve comfort and reduce the risk of causing damage to participants, particularly in those with diabetes where neuropathy may have affected sensation at the foot.

To validate the complete system performance we compared measurements from each sensor against gold standard temperature measuring equipment. Each temperature sensor was placed on a glass plate and surrounded by two reference temperature sensors (Minco S3230) and connected to temperature measurement equipment (NI-9219). We heated the glass plate to at least 20 discrete temperatures over the range 22–42 °C (covering the range of temperatures measured at the foot) and compared the temperature recorded by each sensor against the two reference temperature sensors. Each sensor was measured over a 24-h period, and then repeated three times for each sensor over a period of three weeks. Within this, we were able to quantify the accuracy of the sensors as 0.3 °C, derived from measuring the largest temperature deviation from the gold standard temperature measurement. With this validation protocol repeated over three weeks, the accuracy measurement captures both the worst-case drift (as each sensor was tested multiple times over an extended time period) and systematic/random errors of the sensors.

**Figure 3:**
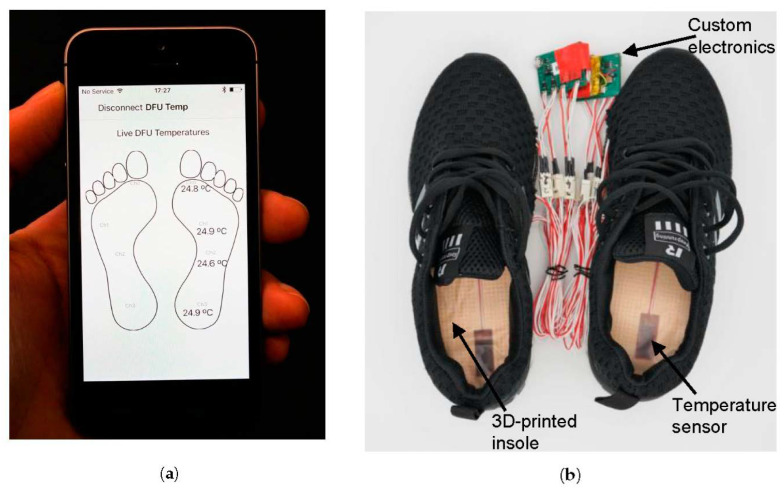
New wearable sensor insole system developed in this work for the continuous monitoring of plantar foot temperatures. (**a**) The iPhone app used for real-time visualisation and logging of data; (**b**) The smart insole system used by participants with the devices fitted inside shoes.

The complete system performs with a precision of 0.036 °C, accuracy 0.3 °C, sample rate 1 Hz and temperature response time constant of 19 s. An iPhone app was also developed to collect the data from the insole devices, shown in Figure 3a. We refer to this device as the smart insole system in this paper, with the complete system shown in Figure 3b where it is configured for a participant.

### 2.2. Participants

A total of 25 participants were recruited to take part in an exploratory study to investigate the temperature dynamics at the foot in participants with diabetes. Participants were recruited through advertisements at the University of Manchester (rather than a hospital clinic or diabetes support group) and invited by email. Given the exploratory nature of this study, participant numbers were chosen by convenience sampling. Participants were split into two groups, a control group with no diagnosis of diabetes (C) and a group with diabetes but no peripheral neuropathy (D). The participant demographics are detailed in Table 1.

Participants were excluded from the study if they reported any foot conditions which included: history of foot ulceration, a major or minor foot amputation, peripheral arterial disease, charcot foot, damaged skin tissue on their feet, any other skin conditions associated with the diabetic foot as well as severe eczema or skin allergies. Participants were tested for lack of sensation in their feet with the 10 g monofilament test at the hallux, first and fifth metatarsal heads and calcaneus [4]. Any participants who demonstrated a lack of sensation in this test were not invited to take part in the study (zero participants).

### 2.3. Experimental Protocol

**Figure 4:**
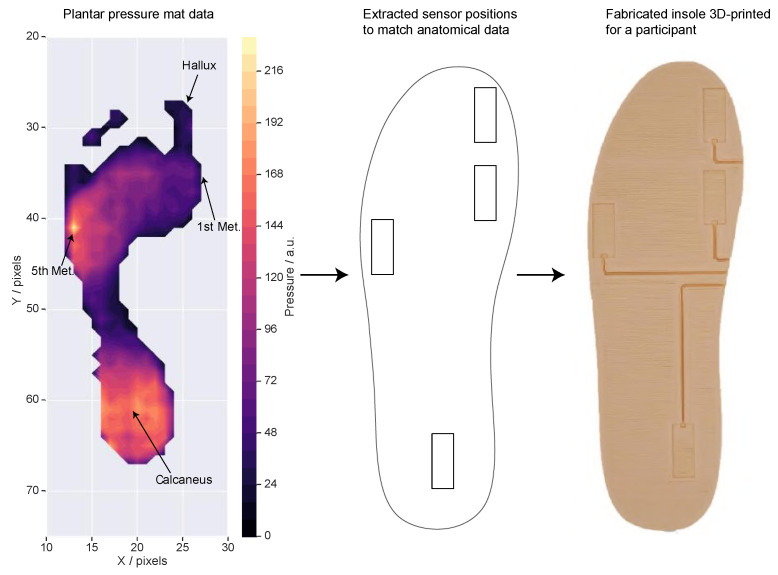
The process to generate a personalised insole for a participant, showing the steps to convert the plantar pressure mat data into a 3D-printed insole.

The experimental protocol consisted of two sessions. The first was used to inform the sensor placement by collecting anatomical information from the participant’s feet. This was undertaken by instructing participants to use a plantar pressure mat (HR Mat, Tekscan Inc., Boston, USA), sampling at 128 Hz for 8 s. Each participant was asked to sit in a chair in front of the mat and align their feet with an insole outline matching their shoe size. Participants performed four stances: standing stationary on both feet, standing stationary on their left foot, standing stationary on their right foot, and rocking back and forward on both feet. The data from the pressure mat was used to generate customised 3D-printed insoles for each participant, containing mounting points for the temperature sensors which were matched to the anatomical landmarks for that participant. The anatomical landmarks were manually identified by overlaying the pressure data over the insole outline, as shown in Figure 4. The centre of the sensors was placed on the area of the highest pressure near the desired anatomical landmark. In some cases, this would lead to the edges of the sensor being outside the insole. These cases were manually altered until the edges of the sensor were inside the insole. In all cases, the sensors were still covering the identified landmark.

**Figure 5:**
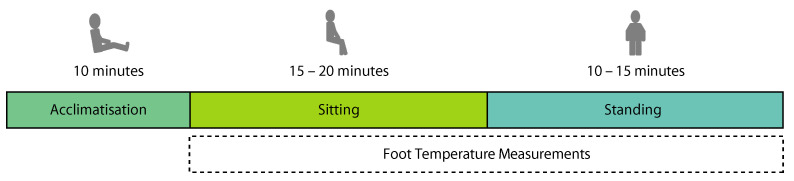
Experimental protocol for the second session of the experimental protocol which was used to collect the temperature data.

The second session was used to collect temperature data from the participant, with the insoles printed between the two sessions (mean of 37 days between sessions). The insoles were fitted in synthetic knitted running trainers (Kuako Keep Running, Amazon.com, Inc., Washington, DC, USA) matching the participant’s reported shoe size and connected to the smart insole system described in Section 2.1. The temperature of the room was logged using a Digital Multimeter and a K-type thermocouple (34465A, Keysight Technologies, Santa Rosa, CA, USA), precision 0.01 °C, accuracy 2.5 °C. The temperature of the room was regulated using a fan heater to keep temperatures consistent between participants, with the temperature during testing sessions recorded as 23.70 ± 1.01 °C (mean ± SD). At the start of the second session, participants performed a 10-min acclimatisation phase, sitting barefoot with their feet reclined to allow the temperature of their feet to stabilise. Participants were then asked to place their feet inside the shoes containing the smart insole system, which was set to start recording. Participants were asked to sit stationary with their legs at an approximate 90° angle at the knee joint for a period between 15–20 min. Following this, participants were asked to stand stationary (while still wearing the shoes and sensing insoles) for a period between 10–15 min. This protocol is summarised in Figure 5.

### 2.4. Data Analysis and Model Fitting

**Figure 6:**
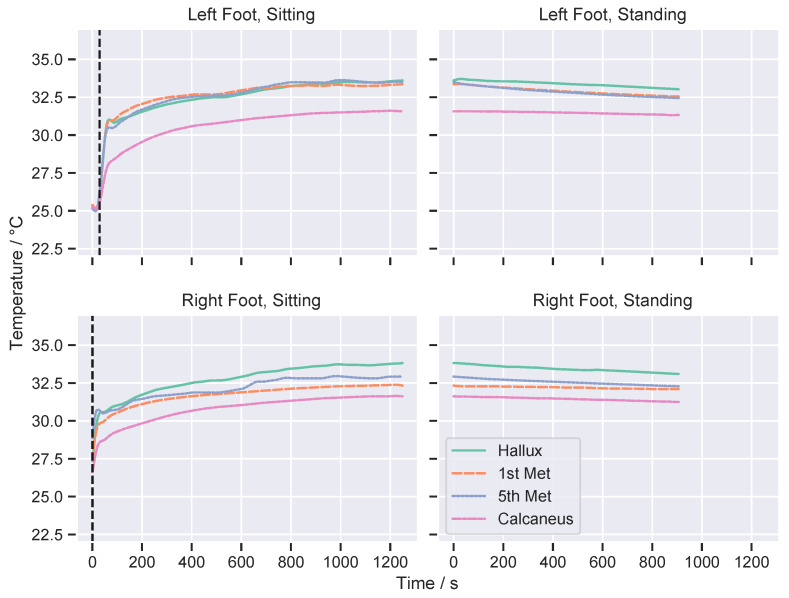
Example raw data from a single participant, showing data for both feet in the sitting and standing stages. Vertical dashed lines indicate the point when the participant put the shoes on. Note that the standing period immediately follows the sitting period (without a break) and therefore the data is continuous between the sitting and standing panels in this plot.

All data were analysed in Python 3.7 using the Anaconda Distribution v2019.03. The data was filtered with an eighth-order zero-phase Butterworth low-pass filter with cut-off frequency of 0.02 Hz, to remove quantisation noise. This cut-off was selected as a value low enough to remove the quantisation noise while sufficiently far below the time constants seen at the foot in this study. Initial analysis of the data found that the temperature of each sensor followed a general trend; once the shoes were worn the temperature change followed an increasing exponential decay function with most participants not reaching a steady-state temperature within the sitting period. When the participants stood up, generally the temperature of each of the sensors tended to decrease, following an approximately linear straight line and again did not tend to reach steady-state within the duration of the standing period. The general trend of this is shown in Figure 6 for an illustrative participant.

To allow comparison of these dynamic temperature measurements between participants, the temperature data from each sensor during sitting and standing was fitted to two mathematical functions which describe the general trends seen in the data.

During the sitting period, the data for each sensor, for each participant, were fit to an increasing exponential decay function described in (1),

(1) $$y\left(t\right)=b+A\left(1-e^{-t/\tau }\right),$$

where *b* is the initial temperature of the foot, *A* is the change in temperature of the foot during the mathematical fitting period, τ is the time constant and y(t) is the temperature at time *t*. Data were fit using non-linear least squares to the middle section of the sitting period, removing 240 s from the beginning and end of the sitting record to remove both the effect of the system itself reaching steady-state (which takes around 95 s) and the effect of noise at the end and the start of the record as some participants became restless, leading to more motion artefacts to be seen. Bounds on the fit were provided, limiting the initial value (*b*) within ±0.02 °C of the temperature at the start of the fitting period (for this specific sensor), and bounding the time constant (τ) between 0 and 1500 s. All values with a poor fit ($r^{2}<0.98$) were excluded from the analysis. Finally, all fits that gave impracticably long times to reach steady-state (τ > 1000 s) were discarded.

During the standing period, the data for each sensor, for each participant, was fitted to a straight line function described in (2),

(2) y(t)=mt+c,

where *m* is the gradient of the line, *t* is time, *c* is the initial fitted temperature, and y(t) is the measured temperature at time *t*. Again non-linear least squares were used to fit the function and the function was fit near the beginning of the standing period, 60 s after the participant had stood up with a total analysis time of 120 s.

We also calculated the absolute temperature differences between sites on the two feet. These are calculated during the sitting period of the experiment, taken at the time 2τ (in (1)). The difference between the temperature on the left and right foot was calculated at this point, and then the absolute (magnitude) of this value was found.

### 2.5. Statistical Analysis

Tests were performed using the stats functions in the SciPy library (Anaconda Python v2019.03), with α = 0.05. As an exploratory analysis, we did not correct *p* for multiple comparisons, and therefore results should be interpreted with due caution. Datasets were initially tested for a Gaussian distribution using the Shapiro–Wilk and D’Agostino-Pearson tests. Given the relatively small number of participants in this study, many of the resultant datasets were not Gaussian, or if one group was Gaussian the other was not. Therefore, we made use of the non-parametric Mann–Whitney U test. We note that non-parametric tests provide less statistical power than parametric tests, therefore providing less confidence that identified differences are actually statistically different and not due to chance alone. The use of non-parametric tests is a limitation of this work but was required due to the non-Gaussian distribution of the data. Tests for significance were performed between the time constant (τ) during sitting, the rate of change (*m*) during standing and the absolute temperature differences. For statistically different results, effect sizes were calculated using Cohen’s d, using the levels for small, medium and large effect sizes of >0.2, >0.5 and >0.8 respectively [36].

## 3. Results

Table 2 shows the absolute temperature differences between feet at each site. Differences in absolute temperatures between groups were small, with participants with diabetes having marginally higher mean temperature differences at all sites apart from the calcaneus. When comparing absolute temperature differences, no significant differences were found between groups at any of the sites.

Table 3 gives the values for the time constants (τ) during sitting at each location, with participants with diabetes having faster time constants compared to the control group, except at the 1st metatarsal head where differences were small. When comparing time constants, significant differences were seen at the hallux and 5th metatarsal head between groups, corresponding to a medium effect size.

The rates of change in temperature during the standing period are shown in Table 3, calculated from the gradient (*m*) of the straight-line function. In general, the temperature of each site decreases during the standing period, indicated by the negative gradient, except for group D at the calcaneus site, which shows a small, but positive, gradient. No significant differences were found for the rate of temperature change during standing.

Some fitted parameters were discarded before analysis due to poor fitting with the model. During the sitting stage, a total of 32 time constants out of 200 were excluded from the analysis (16% of the data, 14.5% from the control group, 17.3% for the diabetes group). During the standing stage a total of 31 gradients from a possible 200 were excluded (15.5% of the data, 14.6% from the control group, 16.3% for the diabetes group).

## 4. Discussion

Foot temperature rise times, identified by extracting time constants, are significantly faster during sitting, at the hallux and 5th metatarsal head, in participants with diabetes compared to controls. To our knowledge, this is the first quantification of the temperature dynamics of the foot between participants with diabetes and controls. The general trend of results shown here are in line with those identified by Niemann et al. [30], who showed similar trends of an exponential rise during sitting followed by a decrease when standing in both participants with diabetes and their control group. Niemann et al. [30] used a longer protocol, with participants sitting and standing for multiple repeated periods, as opposed to a single sitting and standing transition used in this study. However, the authors did not extract fitted parameters on the data such as time constants to allow a quantitative comparison. We note that differences were not seen during the standing stage of our study, which is likely a result of the standing phase following a reasonably long sitting period, and therefore the rate of temperature change during standing is measuring a different biological parameter of the participant, rather than the effect of temperature change after shoe donning. It is possible that if the participant began the study with a standing period rather than a sitting period immediately after putting the shoes on, differences would be seen in the rate of temperature change during standing. The general trend of the data seen here is also in line with those demonstrated by Leister et al. [31]. Leister et al. demonstrated slow exponential rises in temperature during sitting, that did not reach a steady-state value within their experimental protocol. While the protocol is different, Leister et al. identified slower temperature rise times in participants with diabetes with amputation (during walking) compared with diabetes but no amputation. This suggests a pathway where temperature rise times get faster with the development of diabetes, and then potentially decrease again after an amputation. However, the protocols between Leister et al. and this study are different, which is a substantial factor in how temperature dynamics manifest. In particular, Leister et al. identified differences demonstrated during walking rather than sitting. Further, Leister et al. did not identify differences during their sitting period, but this is possibly due to not having a long enough acclimatisation period before the start of the data collection. There is also no comparison to healthy controls, or consideration of personalisation of the sensor placement.

The dynamic temperature differences identified in participants with non-neuropathic diabetes suggest that there are differences in temperature at the foot that are identifiable even before the development of neuropathy, or before displaying differences in temperature asymmetry. This is in contrast to other studies which examine static temperature differences (rather than the continuous data by extracting time constants) in groups who have already developed neuropathy [27,30]. The potential demonstration of much earlier temperature difference between groups is a new contribution here, indicating the potential use of temperature rise time to inform our understanding of DFU development. Temperature monitoring for DFUs focuses on a single snapshot of temperature differences [27,37,38], which may not represent the more complex dynamics of temperature change present in those with diabetes. We highlight that we did not identify differences in the absolute temperatures but did identify differences in dynamic temperatures, captured by extracting the time constant. The dynamic temperature differences could be related to the changes that occur during diabetes onset and progression, such as the changes in soft-tissue biomechanics, where higher shear and elastic moduli are seen in diabetic plantar foot tissue [39], and the vascularisation that occurs from microcirculatory changes through the progression of diabetes [40].

Stratifying clinical grouping could show the stronger significance of temperature rise time differences. In this study, all participants with a diagnosis of diabetes were grouped without stratifying for severity or date of diagnosis. As some participants may have recently been diagnosed or have well-controlled diabetes, they may exhibit a much smaller effect on temperature measurements. In contrast, a participant who has had diabetes for a substantial period of their life or has poor control over their diabetes, may show a larger effect. Therefore, grouping all these participants could limit or exaggerate the size of the effects seen here. However, we note that many participants in this study reported that they did not have any substantial complications from their diabetes. In addition, in this study, we did not control for the tightness of the shoelace. It has been shown that both laces that are too tight and too loose can lead to an increase in temperature [41], and this may have had an impact on the results presented here. Not controlling for tightness of shoelaces is taken as a limitation of this study.

Personalised anatomical sensor positioning through 3D-printing was utilised to develop the device in this study. To the best of our knowledge, no other temperature sensing works in the literature personalise temperature measurement location to account for differences in anatomy. Personalised medicine is an important trend in the academic literature [42,43] and evolving diabetic foot treatment guidance [35]. It comes at the cost of the requirement to collect anatomical information, additional design stages and increased manufacturing time, but may be more effective at detecting the onset of a potential DFU, as sensing can be focused on the key sites of interest. Additionally, given the personalisation aspects presented here, it may also be possible for clinicians to pre-determine the site of a potential DFU using our device, as the sensing is localised to individual areas on the foot. It would be beneficial to compare the results here with those in [30] using the same protocol other than the personalisation, to demonstrate its utility and if the identification of significant differences prior to ulceration highlighted in this paper were a result of the personalised design.

## 5. Conclusions

This paper has presented a new wearable sensor insole for the monitoring of dynamic temperature changes at the foot. 3D-printing was utilised to personalise the insole fit to each user placing sensors at key anatomical locations. While the results are at the proof on concept stage, they suggest that temperature rise times may inform our understanding of DFU development risk, especially as these differences were identified in this study with participants who had no neuropathy. This study suggests that temperature rise time measured dynamically in-shoe at the plantar surface could be a new biomarker related to differences in soft tissue biomechanics and vascularisation during diabetes onset and progression. Our early results show that temperature rise times are faster in participants with diabetes than in healthy controls. We thus recommend more research is undertaken to investigate temperature dynamics, specifically temperature rise times at the foot, in participants with diabetes, which could be linked to the precursors of risk of ulcer development.

## Figures and Tables

**Table 1:** Demographics of participants in this study. Control group participants (C) are those with no diagnosis of diabetes, participants with diabetes (D) only include those with no peripheral neuropathy. Values are mean ± standard deviation. Body mass index (BMI).

	C	D
Number of participants	12	13
Gender (female/male)	5/7	7/6
Age (years)	42.1 ± 17.7	44.4 ± 22.8
Height (cm)	172.1 ± 8.8	169.7 ± 7.7
Mass (kg)	76.9 ± 17.1	77.4 ± 16.6
BMI (kg/m^2^)	25.6 ± 3.6	26.9 ± 5.9

**Table 2:** Values of temperature differences between groups during the sitting stage. Control group participants (C), participants with diabetes (D). Values are mean ± SD. * *p* < 0.05.

Site	Temperature Difference/°C		*p*
	C	D	
Hallux	1.19 ± 0.83	1.33 ± 1.39	0.393
1st Metatarsal Head	0.56 ± 0.49	0.60 ± 0.42	0.388
5th Metatarsal Head	0.76 ± 0.69	0.77 ± 0.34	0.298
Calcaneus	0.62 ± 0.75	0.57 ± 0.58	0.477

**Table 3:** Results from the sitting and standing stages. Control group participants (C), participants with diabetes (D). Values are mean ± SD. * *p* < 0.05.

***Sitting***				
**Site**	**Time Constant (*τ*)/s**		***p***	**Effect Size**
	**C**	**D**		
Hallux	370.4 ± 172.1	279.1 ± 154.1	0.032 *	0.55
1st Metatarsal Head	322.3 ± 200.9	329.6 ± 134.9	0.299	–
5th Metatarsal Head	481.9 ± 225.4	356.6 ± 165.0	0.011 *	0.62
Calcaneus	483.9 ± 148.6	422.5 ± 189.7	0.081	–
***Standing***				
**Site**	**Rate of Temperature Change (m)/$\frac{m{\;}^{{}^{\circ}}C}{s}$**		***p***	**Effect Size**
	**C**	**D**		
Hallux	−0.87 ± 1.54	−0.80 ± 1.50	0.430	–
1st Metatarsal Head	−0.77 ± 1.06	−0.97 ± 1.22	0.324	–
5th Metatarsal Head	−0.74 ± 1.11	−0.91 ± 1.18	0.299	–
Calcaneus	−0.07 ± 0.79	0.00 ± 1.18	0.286	–

## Data Availability

The data presented in this study are openly available in Mendeley Data at https://doi.org/10.17632/ppwxdgbbx4.1, accessed on 15 February 2021.

## Abbreviations

The following abbreviations are used in this manuscript:

ADC	Analogue-to-Digital Converter
BLE	Bluetooth Low Energy
BMI	Body-Mass Index
DFU	Diabetic Foot Ulcer
EEPROM	Electrically Erasable Programmable Read-Only Memory
IA	Instrumentation Amplifier
LDO	Low Dropout
RTD	Resistance Temperature Detector
SD	Standard Deviation
